# A review of the psychometric properties and implications for the use of the fertility quality of life tool

**DOI:** 10.1186/s12955-023-02125-x

**Published:** 2023-05-12

**Authors:** Brittany M. Woods, Leigh Ann Bray, Sukhkamal Campbell, Aimee Holland, Sylvie Mrug, Sigrid Ladores

**Affiliations:** 1 University of Alabama at Birmingham School of Nursing, 1720 2nd Avenue South, NB482, AL 35294-1210 Birmingham, USA; 2 University of Alabama Capstone College of Nursing, Office 3160, Box 870358, Tuscaloosa, AL 35487 USA; 3 Fertility Preservation Services, University of Alabama at Birmingham Medicine, 1700 6Th Ave South, Women and Infants Center 10390, Birmingham, AL 35233 USA; 4 Graduate Clinical Education, University of Alabama at Birmingham School of Nursing, 1720 2nd Avenue South, NB 406A, Birmingham, AL 35294-1210 USA; 5 University of Alabama at Birmingham, 1720 2nd Ave South, CH415, Birmingham, AL 35294-1170 USA; 6 University of Alabama at Birmingham School of Nursing, 1720 2nd Avenue South, NB422, Birmingham, AL 35294-1210 USA

**Keywords:** Infertility, Quality of life, Systematic review, Psychometrics

## Abstract

**Objectives:**

To analyze and synthesize the reported psychometric properties of the Fertility Quality of Life (FertiQoL) instrument and describe its implications for use in practice and research in men and women with infertility.

**Methods:**

A systematic literature search was performed to identify all articles using the FertiQoL tool. PubMed, CINAHL, and PsycINFO were searched from September 2006 through May 2022. Studies were eligible for inclusion if they reported psychometric data on the original FertiQoL tool using a sample population of individuals with infertility. Sample size, country of origin, and psychometric data were documented for each study.

**Results:**

The initial search revealed 153 articles that had utilized the FertiQoL. Following abstract, title, and full-text screenings, 53 articles reported psychometric data and met criteria for inclusion. The FertiQoL is a sound measurement with satisfactory reliability and validity. Studies indicated adequate reliability in the overall scale ($\alpha=0.43-0.92$), as well as the core Emotional, Mind/Body, Social, and Relational scales ($\alpha=0.43-0.92$) and two optional Tolerability and Environment fertility treatment subscales ($\alpha=0.67-0.91$). Although the Relational subscale exhibited slightly lower reliability in several studies, the internal consistency for the measurement as a whole was satisfactory. Results also indicate adequate: 1) face and content validity with extensive professional and patient feedback during development; 2) convergent validity with general quality of life, depression, and anxiety measurements; and 3) structural validity using both confirmatory and exploratory factor analyses.

**Conclusion:**

The FertiQoL tool is the most commonly used instrument to measure the impact of fertility issues on quality of life in men and women with infertility. Understanding the impact of infertility on quality of life provides valuable insight into the areas of infertility-related care that need to be prioritized, such as mental health or relational stressors. While the instrument has been used in different patient populations with infertility and available in multiple translations, it is necessary to understand the updated psychometric properties and the implications for its use. This review shows that the FertiQoL is reliable and valid for cross-cultural use among individuals with various etiologies of infertility.

## Introduction

Between 2006 and 2010, prevalence studies estimated that approximately 72–186 million individuals worldwide were affected by infertility [1, 2], a reproductive disease that results in the inability to conceive after 12 months of unprotected sex [3, 4]. Despite the immense number of individuals that are affected globally, the vast social, physical, and mental health implications of infertility have been largely unaddressed in the last 15 years [5]. Infertility can be female-specific, male-specific, or a combination of various factors and etiologies [6]. Female-specific factors can include endometriosis, diminished ovarian reserve, and polycystic ovarian syndrome [6], while male-specific infertility can evolve from poor sperm quality, quantity, or medical comorbidities [7].

Regardless of the etiology, individuals and couples with infertility face significant infertility-related stress stemming from life-changing decisions regarding their path to parenthood or to remain childless. Individuals with infertility report symptoms of anxiety and depression at rates between 25 and 60%, similar to those with chronic health conditions [8], while approximately 2.7–9.5% of individuals in the general population experience anxiety and depression [9–12]. In addition to the psychological distress of infertility, financial burdens of infertility treatments and patient comorbidities can further limit reproductive options, further compounding infertility-related stress and creating additional barriers to parenthood [13]. Previous studies on infertility-related mental health have historically focused on general infertility or those pursuing assisted reproductive technologies (ART). However, the mental health of subpopulations of people with infertility, such as individuals who choose not to or cannot afford to pursue ART, those with non-anatomical causes of infertility, such as diminished ovarian reserve, or those with iatrogenic infertility following radiation and chemotherapy treatments for cancer, have been largely understudied.

Understanding the impact of infertility on quality of life provides valuable insight into the areas of infertility-related care that need to be prioritized, such as mental health or relational stressors. Three of the most commonly utilized instruments for assessing patient-reported outcomes related to fertility quality of life include the Fertility Problem Stress (FPS) questionnaire [14], the Fertility Quality of Life (FertiQoL) questionnaire [15], and the Fertility Problem Inventory (FPI; [16]).

The FPI is a self-report questionnaire that examines the impact of infertility-related stress in individuals with infertility. The FPI provides a global score by combining five domains determined to be most relevant to those with infertility: 1) social concern, 2) sexual concern, 3) relationship concern, 4) need for parenthood, and 5) rejection of a childfree lifestyle [16]. The FPI is available in 11 languages and contains 46 questions, but is currently only available in paper format, requiring an administrator to convert the survey into an electronic format using survey software, if desired [17]. Alternatively, the FPS is a self-report questionnaire with only 14 items [14]. While the participant burden may be lower compared to FPI with fewer questions to answer, the FPS is also limited to a paper format that would require electronic conversion, and has only been translated into two languages. Neither the FPI nor the FPS creators have reported data on the instrument validation process, or have indicated that input from infertile patients was sought during the development of their measures [17]. In addition, rather than assessing the impact of infertility on a person’s quality of life, these measures focus on the concept of infertility-related stress. The FertiQoL questionnaire is currently the most widely used instrument for measuring fertility quality of life in individuals with infertility. However, there has been a sharp increase in the number of studies utilizing the FertiQoL instrument in the last several years. An updated review is needed to provide researchers and providers with the most current evidence on the utility and soundness of the FertiQoL. This review aims to analyze and synthesize the reported psychometric properties of the FertiQoL instrument and describe implications for its use in practice and research.

## Methods

### Search strategy

A literature search was performed to identify research studies that used the FertiQoL questionnaire. The search was completed on May 4^th^, 2022, using PubMed via the National Library of Medicine, CINAHL through the EBSCOhost platform, and PsycINFO using ProQuest. No additional articles were identified through hand-searching article reference lists using the ancestry approach. No date restrictions were placed on the search to ensure all studies utilizing the FertiQoL were included. However, results included articles published from September 2006 through April 2022. The search strategy for all three databases included keywords “fertility quality of life,” “FertiQoL,” and “fertility-related quality of life,” and application of the “English” filter. Inclusion criteria included: 1) primary research studies; 2) sample population of individuals or couples with infertility; 3) and psychometrics reported on the original FertiQoL instrument. Articles were excluded for the following criteria: 1) secondary research studies or reviews and 2) studies using a modified version of the FertiQoL instrument.

## Results

**Fig. 1:**
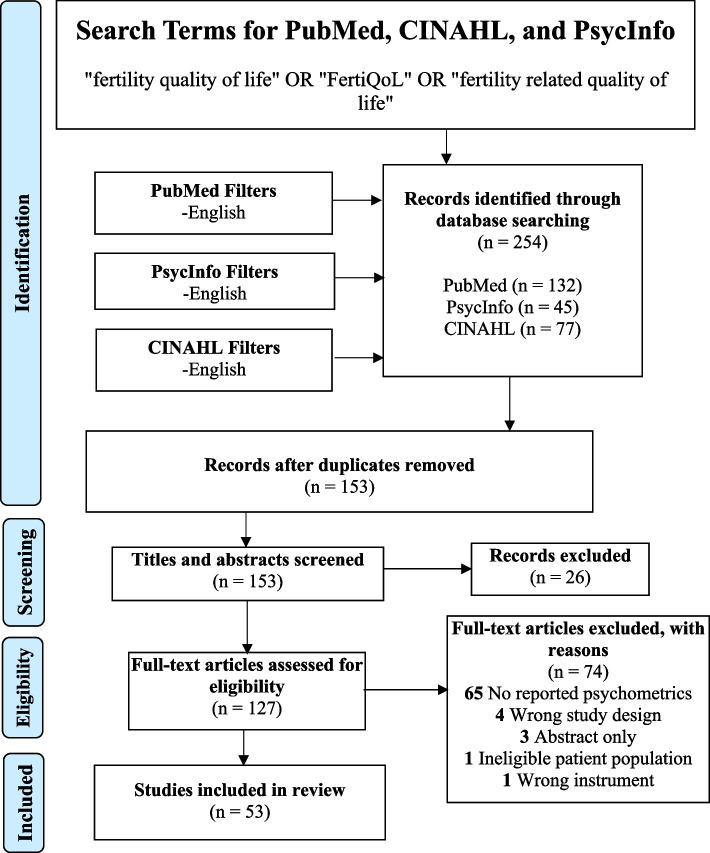
PRISMA diagram for fertility quality of life

One hundred thirty-two articles were initially retrieved from PubMed, 77 from CINAHL, and 45 from PsycInfo, for a total of 254 results. After the removal of 101 duplicates, 153 articles were available to screen. Following title and abstract screening, 26 articles were excluded, leaving 127 for review. Following the inclusion and exclusion criteria, 74 articles were excluded. Sixty-five articles did not report any psychometric properties of the FertiQoL questionnaire in their study sample, four were not empirical research studies (reviews and books), three were only published as abstracts, one included the use of an ineligible patient population, and one did not use the FertiQoL to measure fertility quality of life. Fifty-three articles were ultimately included in the current review (See Fig. 1 for PRISMA diagram).

**Fig. 2:**
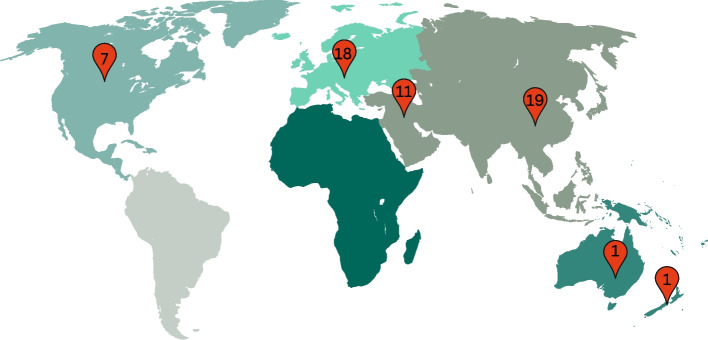
Global disbursement of partic﻿ipants

The majority of the articles collected data using a paper version of the FertiQoL instrument (*n* = 29), followed by online collection (*n* = 10), or a combination of paper and online data collection methods (*n* = 6). Eight articles did not specify whether data collection was completed using the paper or online version. Thirty-three studies were conducted using a female sample, two were male-specific, 11 were female and male dyads, and seven were uncoupled males and females, with an average age of 34.3 across all studies. Twenty-one countries were represented in the study results, with 19 studies originating from East Asia, 18 from Europe, 11 from the Middle East, 7 from North America, and one each from Australia and New Zealand. Additionally, six studies were multisite studies with participants from more than one country. See Fig. 2 for a map of countries represented.

### Fertility quality of life tool development

The FertiQoL was published in 2011 as a 36-item self-report questionnaire designed to measure the impact of fertility problems on quality of life in both men and women suffering from infertility [15]. The development of the FertiQoL was a collaborative effort among the European Society for Reproductive Medicine, the American Society for Reproductive Medicine, and Merck-Serono. It was led by 1) psychology professor and researcher, Jacky Boivin; 2) clinical health psychologist and assistant professor, Janet Takefman; and 3) clinical professor and psychologist, Andrea Braverman [18]. Two questions rate overall quality of life and physical health, 24 core questions assess the impact of infertility on quality of life, and an optional treatment-specific module contains 10-questions for participants pursuing infertility treatments [19]. While it is condition-specific (infertility), it is not specific to underlying causes of infertility, such as endometriosis or polycystic ovarian syndrome. It is acceptable for use in both men and women experiencing infertility, those pursuing treatment, and those who are not. Except for the optional treatment section, the FertiQoL is a static measurement where everyone completes the same number of questions [19, 20].

While no theoretical framework was specified for the development of the FertiQoL, authors mirrored the development protocol of the World Health Organization Quality of Life (WHOQOL) measure that emphasizes quality of life as a multidimensional concept consisting of a person’s perception of their physical and psychological health, level of independence, social relationships, environment, and personal beliefs [15, 21]. The FertiQoL was designed using classical test theory in collaboration with international psychosocial experts in reproductive health and a steering committee [15]. After conducting a literature review to generate an initial pool of 302 items dispersed among 14 domains, the pool was then reduced to 116 items after eliminating redundant and irrelevant items. Seventeen focus groups in five countries were conducted with infertility patients, excluding an additional 14 items, for a total of 102 items. The feasibility and acceptability survey exposed any problematic questions, and the item pool was reduced to the final measurement structure: 24 core items, two overall health items, and ten optional treatment items [15]. Psychometric evaluations, exploratory factor analyses, and factor loadings of the items revealed mostly high reliability and sensitivity for both the subscales and the total scales [15].

### Data collection and scoring

The FertiQoL self-report questionnaire is available in both paper and electronic formats. While free to administer, no alterations can be made to the questionnaire, and creators should be acknowledged in any publication [22]. Scores, sample size, means, and standard deviations should be sent to the FertiQoL authors for publication on their website [22]. The paper format is available in 48 languages, and the electronic is available in 11. The only instructions necessary for completing the survey are: 1) select the response that most reflects how you feel in your current thoughts and feelings, and 2) only complete the questions with an asterisk if you have a partner [19].

Overall, minimal training is required to administer and score the FertiQoL questionnaire. Scoring is automatic when completing the FertiQoL online. Participants can provide a clinic name, identification number, and email address where they would like the results sent. Alternatively, scores must be computed manually or using an Excel algorithm when administering the paper format, with five core and two treatment questions requiring reverse scoring before scaling the raw subscale and total scores. There are two Excel options for scoring the FertiQoL: 1) the researcher can manually enter scores for each participant into the corresponding question box and score it themselves, or 2) scores can be entered for each question, with the Excel algorithm providing the raw and scaled subscale and total scores for each person. Individuals collecting and processing survey data need a basic understanding of Excel and its functions, mainly the ability to input scores into correlating boxes. If participants complete the online version and provide an email address, the results obtained from the online FertiQoL will also be delivered electronically to their email in Excel format. Participant data can then be combined into one Excel datasheet to view answers to individual questions, subscale scores, and total scores within one file.

There are certain risks to privacy that could be encountered when completing the FertiQoL online because individuals are required to provide initials, date of birth, country of residence, and gender, increasing the ability to identify a participant. Without specific protocols preventing the collection of internet protocol (IP) addresses with an electronic survey, individuals may be at an increased risk of privacy breach. However, survey results can be de-identified and protected once data collection is complete. Because of the risk of privacy breach, individuals should be made aware of the measures taken by researchers and clinicians to protect their identity and personal health information.

Scoring the complete FertiQoL, including the optional treatment module, produces six subscales and three total scores [23]. The subscales include four scales with six questions each (Emotional, Mind/Body, Relational, and Social) and an optional module with two subscales looking at environment and treatment tolerability with four and six questions each, respectively. The four required scales comprise the Core FertiQoL score, while the two optional scales make up the Treatment score. These two scores combine to provide the total quality of life score.

Items are measured as continuous variables on a Likert scale that produces a value between zero and four. Likert scale options include very poor (0) to very good (4), always (0) to never (4), and an extreme amount (0) to not at all (4), with some items requiring reverse scoring [23]. Values are summed and scaled to provide subscale and total scores. Both total and subscale scores range from zero to 100, with higher scores indicating better quality of life. While scores are left to interpretation because of a lack of guidelines, the instrument creators provide access to a compilation of published means and standard deviations of total and subscales scores using the FertiQoL tool [24].

### Validity

Validity is the ability of an instrument to accurately measure a construct that it intends to measure [20]. The three main types of validity are content and face validity, criterion validity, and construct validity, with each consisting of several aspects. Criterion validity refers to the degree that scores on a focal measurement adequately reflect that of a gold standard [20, 25]. Since there is currently no gold standard measurement for infertility specific quality of life, criterion validity has not been measured for the FertiQoL and will not be addressed in this review. Rather, this review will report on the content and face validity and construct validity of the FertiQoL.

### Face and content validity

Face and content validity are subjective evaluations that ensure an instrument reflects the construct it intends to measure [20]. Providers and patients can assess face validity to ensure that an instrument appears to measure its intended construct. Face validity is often critical when developing disease-specific measurements, like the FertiQoL, because general measures may not seem relevant to participants, reducing the potential for completion and accuracy of a generalized tool [20, 25]. Alternatively, content validity is usually assessed by field experts, like clinicians and researchers, that ensure the entire construct is being measured [20].

The development of the FertiQoL instrument included extensive integration of results from several focus groups and debriefings comprised of the FertiQoL steering committee and psychosocial reproductive health experts from 11 countries (psychologists, counselors, social workers, researchers, patient user groups, physicians, and nurses), alongside individuals with infertility, where questionnaire items were assessed and deemed both relevant and comprehensive [15, 17]. Vital feedback from the focus groups and debriefings improved face and content validity by correcting wording and eliminating redundant items. An acceptability and feasibility study was also conducted and included 525 participants in 10 countries, with results further supporting prior assertions of face validity and acceptability by individuals with infertility [15].

### Construct validity

#### Convergent validity

Convergent validity is the degree to which scores on a measurement correlate with scores on a measure with which there is a hypothesized relationship [20, 25]. However, in the absence of a “gold standard” measurement, like fertility-specific quality of life, instruments assessing constructs with expected conceptual convergence, like general quality of life, relational satisfaction, anxiety, and depression, may be used instead [20]. To assess convergent validity using a generic quality of life instrument, Heredia et al. [26] used Spearman’s rho (ρ) to measure correlations between the Short Form 36 (SF36) questionnaire for general physical and mental health and the FertiQoL, whereas Hekmatzadeh et al. [27] used the shorter adaptation of the instrument, the 12-item Short Form Health Survey (SF-12) and Pearson’s *r*. Correlations were considered weak ($$<.3)$$, moderate ($$\ge .3<.7)$$, or strong ($$\ge .7),$$ and statistically significant at *p* > 0.05.

The Core and Total scores of the FertiQoL were moderately positively associated with social functioning and mental health subscales of the SF-36 [26]. Both instruments (SF-12 and SF-36) exhibited agreement with moderate positive correlations between the FertiQoL Emotional subscale and mental health, role limitations from emotional problems, and vitality. Additionally, the SF-36 indicated a moderate positive correlation with social functioning. However, the FertiQoL Social and Mind/Body subscales showed more correlational variability with the two adaptations of the Short Form Health Survey, with the SF-12 exhibiting stronger convergence with the FertiQoL Social subscale and between the Mind/Body subscale and physical problems than the SF-36. More specifically, results from the SF-12 indicated a moderate positive correlation between the Social subscale of the FertiQoL and the social functioning domain (*r* = 0.49, *p* < 0.001), while the SF-36 found no significant correlation with the social domain (*ρ* = 0.117), but rather, a moderate positive correlation between the FertiQoL Social subscale and the SF-36 general health domain (*ρ* = 0.360, *p* < 0.05). Additionally, there was a moderate positive correlation between the Mind/Body subscale and role limitations from physical problems (*r* = 0.47, *p* < 0.001) and physical functioning (*r* = 0.68, *p* < 0.001) with the SF-12, but no significant correlations were found with physical functioning (*ρ* = 0.080), physical role limitations (*ρ* = 0.127), or bodily pain (*ρ* = 0.256) on the SF-36. However, results did suggest moderate correlations between the Mind/Body subscale and social functioning (*ρ* = 0.497), mental health (*ρ* = 0.524), vitality (*ρ* = 0.417), and emotional role (*ρ* = 0.417) on the SF-36. Although the two studies vary in correlational significance on certain subscales, overall results provide evidence of adequate convergent validity between measurements of general quality of life and the disease specific FertiQoL.

Since depression and anxiety are two well-known consequences of infertility, the Hospital Anxiety and Depression Scale (HADS; [28]) is often used to confirm convergent validity using correlation coefficients [29]. It has been utilized in multiple populations, including Iranian [27, 30], Turkish [31, 32], and Dutch women with infertility [33]. As hypothesized, significant negative correlations were found between the core total and subscales of the FertiQoL and HADS-Anxiety (HADS-A) and HADS-Depression (HADS-D) scales, with fertility quality of life increasing as depression and anxiety decrease. Weak to moderate associations have been found between the Relational subscale and the HADS-A (*r* = -0.2 – -0.49) and HADS-D (*r* = -0.32 – -0.50). Similar results have been found between the Relational subscale and multiple measurements of relationship quality. In a validation study, Donarelli et al. [34] found weak to moderate positive correlations between the FertiQoL Relational subscale and marital satisfaction (*ρ* = 0.31–0.36) and dyadic adjustment (*ρ* = 0.28–0.31), while moderate negative associations were found with sexual stress (*ρ* = -0.48) and marital commitment (*ρ* = -0.30 – -0.37). All other core subscales had moderate correlations with anxiety and depression. Moderate correlations exist between the core total and HADS-A (*r* = -0.56 – -0.64) and HADS-D (*r* = -0.51 – -0.67). Moderate correlations were reported for the Mind–Body subscale with the HADS-A (*r* = -0.48 – -0.65) and HADS-D (*r* = -0.38 – -0.66), the Social subscale with the HADS-A (*r* = -0.44 – -0.55) and HADS-D (*r* = -0.46 – -0.56), and the Emotional subscale with the HADS-A (*r* = -0.50 to -0.62) and HADS-D (*r* = -0.49 to -0.54). See Table 1 for a summary of correlation coefficients from the studies reporting on the HADS and FertiQoL convergent validity.

**Table 1:** **Table 1 **Pearson's correlations between FertiQoL and HADS

FertiQoL Subscales	HADS-Anxiety					HADS-Depression				
	CORE	MB	REL	SOC	EMO	CORE	MB	REL	SOC	EMO
Aarts, van Empel [33]	-0.64^**^	-0.65^**^	-0.29^**^	-0.48^**^	-0.58^**^	-0.67^**^	-0.66^**^	-0.37^**^	-0.54^**^	-0.54^**^
Dural, Yasa [32]	-0.62^*^	-0.64^*^	-.027^*^	-0.44^*^	-0.56^*^	-0.65^*^	-0.65^*^	-0.35^*^	-0.52^*^	-0.51^*^
Kahyaoglu Sut and Balkanli Kaplan [31]	-0.56^***^	-0.48^***^	-0.20 ns	-0.45^***^	-0.62^***^	-0.51^***^	-0.38^**^	-0.32*	-0.46^***^	-0.49^***^
*Maroufizadeh, Ghaheri *[30]	-0.63^***^	-0.58^***^	-0.49^***^	-0.55^***^	-0.50^***^	-0.66^***^	-0.62^***^	-0.50^***^	-0.56^***^	-0.53^***^

FertiQoL subscales: *CORE* Core total, *MB* Mind/Body, *REL* Relational, *SOC* Social, *EMO* Emotional, *ns* not significant

^*^ *P* < 0.05, **P < 0.01, ***P < 0.001

#### Structural validity

Structural validity is a measurement of how well an instrument captures the hypothesized dimensionality of a complex construct using multiple subscales [20]. Structural validity is most commonly assessed using confirmatory factor analyses (CFA) or exploratory factor analyses (EFA). During the development of the FertiQoL, authors used EFA to explore subscale structure and corroborate the conceptual model [15, 20]. Aside from Hekmatzadeh et al. [27], subsequent studies used CFA to assess structural validity [20]. Donarelli et al. [34] reported a CFA using chi-square, comparative fit (CFI), goodness of fit (GFI), and root mean square error of approximation (RMSEA) indices for the FertiQoL with a good fit for the four-factor model and Relational subscale in 589 infertile Italian men and women. Maroufizadeh et al. [30] also used CFA, reporting chi-square, CFI, RMSEA, and standardized root mean square residual indices to determine goodness of fit of the Persian FertiQoL using a sample of 155 infertile Iranian women. Both studies confirmed goodness of fit with acceptable factor loadings on all items except for one question asking whether infertility had strengthened partner commitment [30, 34]. Alternatively, Hekmatzadeh et al. [27] confirmed the six underlying factors present in the complete Iranian version of the FertiQoL tool (Emotional, Mind/Body, Relational, Social, Environmental, and Tolerability) with a sample of 300 women with infertility in Iran. Results from the EFA with principal component factor analysis indicated all factor loadings were greater than 0.30 and all original questions remained. The FertiQoL has demonstrated structural validity, with studies confirming that the subscales adequately reflect the hypothesized underlying factors.

### Reliability

Reliability refers to a measurements ability to provide consistent and stable scores that are free from error or variation after repeated measurements, under different circumstances, by different persons, or using different measurement versions [20]. Efforts to determine the reliability of the FertiQoL are mostly limited to assessments of internal consistency because of the potential for low temporal stability of psychological states [20]. The cycle of hope and despair cycle experienced with each menstrual or treatment cycle failure makes test–retest reliability problematic [8, 20, 35]. However, while a previous review found no evidence supporting the stability of the FertiQoL over time [17], a recent study by Chan et al. [36] investigated decisional conflict, regret, anxiety, depression, and fertility quality of life in 151 women in Hong Kong notified of an unsuccessful IVF cycle (T_0_). Participants completed the questionnaire again during their consultation 2–3 weeks later (T_1_) and finally, three months later (T_2_). Descriptive statistics suggested relative stability over time, with Core scores of 63.99 (T_0_), 64.67 (T_1_), and 63.96 (T_2_), Treatment scores of 62.03 (T_0_), 61.70 (T_1_), and 60.80 (T_2_), and overall FertiQoL scores of 63.34 (T_0_), 63.77 (T_1_), and 62.91 (T_2_). While the FertiQoL shows potential adequate test–retest reliability, additional studies are needed to support the currently limited findings.

### Internal consistency

Internal consistency, a measurement of reliability related to the homogeneity of items on a scale or subscale [20], has been extensively documented in multiple studies and compiled by the original authors on the Fertility Quality of Life website [24], as well as by Koert et al. [37] in a recent systematic review that summarizes the updated psychometric properties of the FertiQoL. Internal consistency has been reported using Cronbach’s alpha coefficients in all studies using FertiQoL. Internal consistency was tested during the generation of the FertiQoL [15, 33] and subsequently in multiple countries to determine the reliability of different translations and use of the measure with individuals of multiple ethnicities and cultures. Internal consistencies were available for populations with infertility in the U.S., Canada, China, Denmark, Italy, Germany, Hong Kong, Hungary, Iran, Japan, Jordan, Korea, Netherlands, Poland, Portugal, Switzerland, Taiwan, and Turkey. See Tables 2 and 3 for updated internal consistencies with a description of the population sample and country of origin.

**Table 2:** **Table 2 **Internal consistency reported by FertiQoL studies with subscales

Authors	Sample	Emotional	Mind–Body	Relational	Social	Core	Environment	Tolerability	Treatment Total	Scale Total
Aarts, van Empel [33]	472 women with infertility in the Netherlands receiving medical assistance for reproduction	.84	.85	.72	.74	.91				
Asazawa, Jitsuzaki [38]	321 men undergoing IVF in Japan	.79	.82	.62	.67		.75	.65		.89
Asazawa and Mori [39]	233 couples (*N* = 466) attending infertility clinics in Japan	.87	.88	.66	.75		.80	.79		.93
Balsom and Gordon [40]	230 women in the U.S. and Canada trying to conceive 12–48 months without medical intervention	.71	.85	.68	0.43	.78				
Boivin, Takefman [15]	1305 women, 109 men with fertility difficulties in Australia, Canada, New Zealand, UK, USA	.90	.84	.80	.75	.92	.84	.72	.81	.92
Donarelli, Lo Coco [34]	301 women, 288 men undergoing ART with primary infertility in Italy	.83	.83	.65 (F = .68) (M = .61)	.70					
Dural, Yasa [32]	389 women undergoing fertility treatments in Turkey	.82	.81	.73	.70	.89				.89
Hekmatzadeh, Bazarganipour [27]	300 women with infertility in Iran	.77	.78	.79	.77		.83	.79		.82
Kahyaoglu Sut H & Balkanli Kaplan P [31]	89 women with infertility in Turkey	.74	.81	.68	.77	.90	.69	.71	.71	.91
Maroufizadeh, Ghaheri [30] and Maroufizadeh, Ghaheri [41]	155 women with infertility in Iran	.82	.82	.64	.750	.91	.67	.64	.69	
Ngai and Loke [42]	135 couples (*N* = 270) with infertility in China	.79	.85	.60	.63	.88				
Pedro, Frederiksen [43]	161 women and 132 men enrolled for IVF or ICSI in 3 Danish clinics	.90	.84	.80	.75		.84	.72		.92
Pedro, Canavarro [44]	265 women and 83 men undergoing evaluation or treatment for infertility in Portuguese clinics	.88	.89	.72	.78		.81	.75		
Sexty, Hamadneh [45]	144 German, 126 Hungarian, 126 Jordanian new fertility patients and partners	Jordan F = .77 M = .81 Germany F = .79 M = .84 Hungary F = .77 M = .67	Jordan F = .85 M = .89 Germany F = .81 M = .82 Hungary F = .83 M = .80	Jordan F = .60 M = .60 Germany F = .64 M = .64 Hungary F = .65 M = .67	Jordan F = .73 M = .75 Germany F = .75 M = .67 Hungary F = .64 M = .61					
Sexty, Griesinger [46]	362 women and 234 men attending a German infertility clinic	F = .83 M = .84	F = .84 M = .83	F = .70 M = .65	F = .68 M = .67	F = .89 M = .87				
Swift, Reis [47]	230 women undergoing infertility treatments in the U.S	.83	.80	.82	.71	.89				
Szigeti, Grevenstein [48]	320 Hungarian women with infertility	.84	.87	.77	.73	.90				
Volpini, Mazza [49]	323 women with fertility problems in Italy	.87	.85	.70	.72	.90	.81	.71	.91	.78
Warchol-Biedermann [50]	250 men seeking first-time fertility evaluation in Poland	.88	.89	.83	.84	.86				

*IVF* In-vitro fertilization, *ICSI* Intra-cytoplasmic sperm injection, *ART* Assisted reproductive technology, *F* Female, *M* Male

**Table 3:** **Table 3 **Reported internal consistency subscale ranges and overall totals

**FertiQoL Internal Consistency: Subscales Only**				
**Authors**	**Sample**	**Subscale and Ranges**		
Amiri, Brassard [51]	185 couples (*N* = 370) with infertility seeking ART services in Canada	Emotional, mind–body, and relational subscales range from F = .73-.86 and M = .69-.85		
Andrei, Salvatori [52]	133 men and women with anatomical and non-anatomical infertility in Italy	Subscales range from .83—.86		
Chan, Lau [36]	151 women with infertility who did not get pregnant following IVF in Hong Kong	Subscales range from .76—.93		
Gameiro, Canavarro [53]	322 women, 111 men with infertility in Portugal	Relational subscale = .70		Tolerability subscale = .75
**FertiQoL Internal Consistency: Totals and Subscales**				
**Authors**	**Sample**	**Subscales**	**Treatment**	**Total FertiQoL**
Cheng, Stevenson [54]	126 women seeking infertility treatment in Taiwan	Core Total = .91	Total =.81	.91
Cserepes, Bugán [55]	270 couples (*N* = 540) attending their first fertility consultation in Germany and Hungary	Total and subscales range from .63—.88		
Domar, Gross [56]	166 women undergoing their first homologous IVF cycle at a Boston-based U.S. infertility clinic	Total and subscale ranges from .75—.93		
Li, Long [57]	108 women attending a fertility clinic in China for their first IVF treatment	Subscales range from .73—.92 (experimental) and .70 and .90 (Control)		Experimental = .94 Control = .93
Li, Luo [58]	253 women with infertility attending a fertility center in China	Subscales range from .74—.86		.91
Li, Jiang [59]	262 women with RPL in China	Subscales range from .78—.85		.81
Renzi, Di Trani [60]	93 childless women in Rome undergoing ART (IVF, IUI, or ICSI)	Total and subscale range from .70—.92		
**FertiQoL Internal Consistency: Overall Tool Total**				
**Authors**	**Sample**			**FertiQoL Total**
Kayabaşi and Yaman Sözbir [61]	120 women in Turkey with primary infertility pregnant through ART in 2^nd^ or 3^rd^ trimester			.94
Kim, Shin [62]	121 couples with infertility with one or more infertility treatments in South Korea			.93
Li, Zhang [63]	498 women with infertility in China undergoing IVF-ET			.93
Shin, Lee [64]	186 women with primary infertility receiving infertility treatment at least once in Korea			.93
Steuber and High [65]	301 women with infertility in the U.S			.93
Ataman, Aba [66]	797 women receiving infertility treatment in Turkey			.92
Kim, Hong [67]	169 women undergoing IVF in Korea			.92
Ni, Tong [68]	137 women with repeated implantation failure in China			.92
Çambel and Akköz Çevik [69]	125 women receiving infertility treatment in Turkey			.91
Donarelli, Salerno [70]	34 counseled and 34 matched non-counseled couples with primary infertility starting their first IUI, IVF, or ICSI in Italy measured before beginning the cycle (T1) and on day of ET (T2)			T1 = .91(F) & .90(M) T2 = .92(F) & .89 (M)
Jing, Gu [71]	768 women with infertility undergoing IVF-ET in China			.91
Jing, Gu [72]	588 women with infertility undergoing IVF in China			.91
Maroufizadeh, Hosseini [73]	180 couples with infertility in Iran			.91
Kahyaoglu Sut and Balkanli Kaplan [31]	89 women with infertility in Turkey			.91
Haemmerli Keller, Alder [74]	109 women with infertility undergoing NC-IVF and cIVF in Switzerland			.89
Ha and Ban [75]	150 couples with infertility in South Korea			.88
Du and Dong [76]	168 couples with infertility (*N* = 336), no children, and more than one ART cycle in China			.86
Yousefzade, Rezaiee Ahvanuiee [77]	180 men and women with infertility in Iran			.86
Balsom and Gordon [78]	58 women with infertility between 12 and 48 months in the U.S. and Canada			.82
Pozza, Dèttore [79]	226 individuals undergoing homologous and heterologous ART in Italy			.81

*ART* Assisted reproductive technology, *IVF* In-vitro fertilization, *F* Female, *M* Male, *RPL* Recurrent pregnancy loss, *ICSI* Intra-cytoplasmic sperm injection, *IUI* Intrauterine insemination, *ET* Embryo transfer, *IVF-ET* In-vitro fertilization embryo transfer, *NC-IVF* Natural cycle in-vitro fertilization, *cIVF* conventional in-vitro fertilization

Previous studies indicated that FertiQoL is generally reliable in diverse populations of men and women with infertility. Internal consistency alpha scores range from 0.43–0.92 for the four subscales included in the Core (Emotional, Mind/Body, Social, and Relational) and 0.78–0.92 for the Core total (combined core subscales). While only some studies reported internal consistency for the optional Treatment module, those indicated moderate reliability with scores ranging from 0.67–0.84 for the Environment subscale, 0.64–0.79 for the Tolerability subscale, and 0.69–0.91 for the overall Treatment total. The internal consistency for the complete FertiQoL total ranges from 0.78–0.94. While no specific rules exist defining satisfactory internal consistency, many agree that an alpha greater than 0.70–0.75 is generally considered acceptable [20, 80].

Overall, the four subscales that make up the core score of the FertiQoL showed moderate to high reliability. The Emotional (Cronbach’s $\alpha$=0.71–0.90) and Mind–Body (Cronbach’s $\alpha$=0.78–0.89) subscales showed high reliability with all alpha coefficients greater than 0.70. Aside from one study reporting low reliability ($\alpha$=0.43) in women with infertility from the U.S. and Canada trying to conceive between 12 and 48 months without medical intervention [40], the Social subscale (Cronbach’s $\alpha$=0.61–0.84; 4/19 studies $\alpha$ <0.70) showed moderate reliability. Additionally, the Relational subscale has shown slightly lower reliability in several studies, with alphas ranging from 0.60 to 0.80 (9/19 studies $\alpha$=0.60–0.68). Furthermore, two studies reported lower reliability of the Relational subscale with men. Donarelli et al. [34] described lower reliability of the Relational subscale in Italian men (0.61 vs. women: 0.68), and Sexty, Griesinger [46] corroborated these results with lower reliability in German men (0.65 vs. women: 0.70), suggesting the need to use caution when interpreting FertiQoL results for this subscale, particularly with men. Despite the slightly lower reliability in the Relational subscale, the internal consistencies reported indicate that the majority of the FertiQoL has demonstrated acceptable reliability, suggesting that the subscale items reliably measure the same underlying latent trait.

### Implications for practice

Currently, the FertiQoL scores are open to interpretation by the individual administering the instrument or those taking the assessment online. Although a previous review found no evidence of test–retest reliability and a lack of clinically important cutoff scores [17], recent studies have suggested that core FertiQoL scores may correspond to clinically significant thresholds, including anxiety (< 55 to 59) and depression (< 51 to 52) in Dutch and Turkish individuals [31, 32], and marital dysfunction (< 74) in Italian men and women with infertility [34]. Healthcare providers, including physicians, physician assistants, nurses, nurse practitioners, and medical trainees (medical students, undergraduate and graduate nursing students), should be educated on the potential impact that infertility can have on an individual’s quality of life. While it was not specifically designed to detect pathological states of anxiety or depression, it can be used to identify individuals experiencing a more severe impact of infertility on their quality of life [15]. Applying this knowledge to clinical practice would expedite identification of those needing further assessment and additional specialty care when warranted. However, while there is some evidence to propose the translation of FertiQoL values to indicate clinically significant anxiety or depression, additional studies are needed to confirm the findings and ranges before implementation in everyday clinical practice.

## Discussion

Findings from this review suggest that among the few available instruments measuring infertility-stress and fertility-related quality of life, the FertiQoL remains the most widely used fertility-specific quality of life measurement with adequate reliability and validity. Extensive feedback from individuals with infertility and reproductive professionals was integrated into the development of this fertility-specific quality-of-life tool [15]. Numerous studies have evaluated the reliability of the FertiQoL in populations of both men and women with infertility from different ethnicities, cultures, and causes of infertility, and except for the Relational subscale, it shows consistently high reliability in the core total, overall total, and remaining subscales. However, given the potential of psychological states to influence test scores, there is no established optimal time to administer the FertiQoL. Defining an appropriate time frame could be done by assessing test–retest reliability. Unfortunately, the cyclical nature of the hope and despair that individuals with infertility experience after each passing cycle can make test–retest analyses difficult [8, 20, 35], with only one study providing sound evidence of stability over time thus far [36]. Additionally, while the FertiQoL provides the most accurate quantitative measurement of the impact of infertility on an individual’s quality of life, it still cannot capture the small nuances of the lived experience of infertility that can only be elucidated using qualitative methodology.

Future research to further improve the FertiQoL should focus on two main concepts: 1) Evaluating its test–retest reliability and 2) Determining clinically significant threshold scores. Test–retest reliability could be evaluated by assessing several groups of individuals with infertility at multiple points throughout a cycle (e.g., person one tested on day three, person two on day six, person three on day nine) and retesting at regular intervals (1–2 weeks or 1 month) or at the same time during the following cycle (e.g., person one at day three again). While failure or success in achieving a pregnancy, either through assisted reproductive technology or naturally, may affect the results of the analysis, this would also be beneficial to understand how these pivotal events can impact the quality of life for those with infertility. This could also provide insight into FertiQoL’s ability to detect change or capture a participant’s true score [20].

While several instruments exist to measure generic quality of life, depression, and anxiety, an infertility-specific measurement allows clinicians and researchers to differentiate the impact of infertility versus general stressors on an individual’s quality of life [15]. The subscales of the FertiQoL provide a more precise determination of problematic areas that can lead to an impaired quality of life, like relational or emotional concerns. Clinicians can use the FertiQoL to identify areas in need of intervention and offer additional support or resources when possible. The FertiQoL can also provide an opportunity to reinforce an open line of communication between clinicians and patients. Individuals who utilize avoidance coping or conceal negative emotions about infertility are more likely to experience feelings of stigma and depression that negatively affect quality of life [67, 71]. An active approach to monitoring patients for infertility-related quality of life conveys a supportive environment where clinicians are open to communication, providing psychosocial resources, and introducing strategies to improve coping mechanisms and communication within an identified support system.

### Strengths and limitations

Extensive efforts were made to include all literature that used the FertiQoL tool and reported psychometric properties. Although no time limit was placed on the search parameters, additional literature may have been missed due to the selection of keywords, chosen databases, and limitations to studies published in English. Additionally, most of the studies included populations with infertility seeking treatment, excluding a critical portion of individuals who chose not to or could not afford to pursue infertility treatment.

Despite the limitations outlined, this report offers several strengths. This is the most recent comprehensive literature review and synthesis of a psychometric evaluation of the FertiQoL. A systematic approach was used to identify studies available in English that reported FertiQoL psychometric properties from three comprehensive databases: PsycINFO, PubMed, and CINAHL. It outlined its implications for use and identified areas in need of further investigation to advance current research on infertility-related quality of life.

## Conclusion

This review demonstrates that the FertiQoL is a sound measurement tool with adequate reliability and validity for use with individuals with infertility from various ethnicities and cultures. With further investigation into clinically significant thresholds, the FertiQoL could be used to reduce patient burden as a single, initial assessment tool in individuals experiencing fertility challenges to identify those needing further assessment and care. Despite the ability of the FertiQoL to ascertain potential areas of infertility-related challenges, like mental health and relational problems, the use of qualitative research methodologies should be considered to fully explore the multifaceted issues faced by people with infertility and identify the best ways to deliver comprehensive clinical care to meet their needs.

## Data Availability

The datasets used and/or analyzed during the current study are available from the corresponding author on reasonable request.

## Abbreviations

ART: Assisted reproductive technologies
CFI: Confirmatory factor analysis
EFA: Exploratory factor analysis
FertiQoL: Fertility quality of life
FPI: Fertility Problem Inventory
FPS: Fertility Problem Stress Questionnaire
HADS: Hospital Anxiety and Depression Scale
IVF: In-vitro Fertilization
SF-36: Short Form 36
SF-12: 12-Item Short Form Health Survey
